# Effective Preservation of Chilled Pork Using Photodynamic Antibacterial Film Based on Curcumin-β-Cyclodextrin Complex

**DOI:** 10.3390/polym15041023

**Published:** 2023-02-18

**Authors:** Jingru Wu, Jing Li, Fang Xu, Arong Zhou, Shaoxiao Zeng, Baodong Zheng, Shaoling Lin

**Affiliations:** College of Food Science, Fujian Agriculture and Forestry University, Fuzhou 350002, China

**Keywords:** curcumin, storage, chilled pork, photodynamic inactivation

## Abstract

A biodegradable photodynamic antibacterial film (PS-CF) was prepared using the casting method, with κ-Carrageenan (κ-Car) as the film-forming substrate and curcumin-β-cyclodextrin (Cur-β-CD) complex as photosensitizer. Chilled pork samples were coated with PS-CF and stored at 4 °C to investigate the effects of PS-CF combined with LED light irradiation (425 nm, 45 min) (PS+L+) on pork preservation during 10 days of storage. The total viable count (TVC) of bacteria, total volatile basic nitrogen value (TVB-N) and the pH of pork treated with PS+L+ were all lower than the control, and the water-holding capacity (WHC) was higher. Ten days later, the TVB-N value was 12.35 ± 0.57 mg/100 g and the TVC value was 5.78 ± 0.17 log CFU/g, which was within the acceptable range. Sensory evaluation determined that the color, odor, and overall acceptability of pork treated with PS+L+ were significantly better than the control. These findings suggest that PS+L+ treatment effectively extended the shelf life of chilled pork from ~4–5 to 10 days. Correlation analysis showed that the sensory quality of the chilled pork significantly correlated with total bacterial counts, TVB-N and thiobarbituric acid reactive substances (TBARS) (*p* < 0.05), suggesting that these biomarkers could be used as standard indicators for evaluating the freshness of chilled pork. These findings demonstrate the effectiveness of Cur-β-CD photodynamic antibacterial film for the preservation of chilled pork and provide a theoretical basis for the application of the film for the preservation of fresh food in general.

## 1. Introduction

Pork is a very popular meat because of its relatively low price, pleasant flavor, tenderness, juiciness and high nutritional value [1]. However, fresh pork has a shelf life of only a few days due to microbial growth and fat oxidation resulting from the high moisture, fat and protein content of the pork, which causes drip loss (i.e., weight loss), reduced quality and lower commercial value [2]. In the food industry, the main methods of preserving chilled pork are low temperature, high-voltage electrostatic field, controlled atmosphere and packaging [3]. Frozen storage is simple, convenient and practical, and can greatly extend the shelf life of pork, but freeze-thawing damages the meat tissue structure, resulting in serious drip loss after thawing [4,5]. High-pressure preservation strongly inhibits the growth and reproduction of microorganisms and does not reduce the original eating quality of pork, but the equipment is expensive and the production cost is high [6]. 

Currently, packaging is the main method for preserving chilled pork and research has focused on enhancing the mechanical properties of packaging materials; antimicrobial packaging has great potential yet has received less attention. Consumer demand for antimicrobial food packaging has increased significantly since the outbreak of COVID-19, so developing practical antimicrobial food packaging has become increasingly urgent [7]. Carrageenan is suitable for the preparation of antibacterial packaging because of its excellent film-forming properties, food grade status, barrier properties and safety, which satisfy the demand for edible food packaging [8,9]. However, the intrinsic antimicrobial effect of carrageenan is weak and insufficient to effectively preserve food from microbial spoilage, so antimicrobial agents are frequently added to carrageenan films to enhance their preservative properties [10]. For example, an edible antibacterial film was prepared by adding Aloe vera extract to carrageenan [11], but the antimicrobial effect was poor, so more effective antimicrobial agents are needed.

Photodynamic inactivation (PDI), a non-thermal sterilization technology, has received widespread attention because of its food acceptability, safety and much lower energy consumption than thermal processing [12]. PDI not only effectively inactivates pathogenic microorganisms, including bacteria, fungi and viruses, but also rarely induces microbial resistance, or genotoxicity [13,14]. The three critical factors that are indispensable for effective PDI are the light source, photosensitizer and oxygen. After the photosensitizer has been activated by an appropriate wavelength of visible light, reactive oxygen species (ROS) are produced by both electron transfer (type I) and energy transfer (type II) processes. ROS damage microbial cellular components—mainly membrane lipids, DNA and enzymes—disrupting their metabolic activity and ultimately inducing programmed cell death [15,16,17]. Therefore, an appropriate photosensitizer is a vital element of PDI.

Curcumin (Cur) is an important edible coloring agent, a polyphenolic compound extracted from the rhizome of turmeric [18] and approved by the Codex Alimentarius Commission and European Food Safety Authority as a food additive. It is extensively used as a food ingredient and in medical applications [19]. Cur is a novel, third-generation photosensitizer with high targeting efficacy. When activated by visible light (400–500 nm), Cur has a strong antimicrobial effect against pathogenic bacteria such as *Acinetobacter baumannii*, *Streptococcus mutans* and *Listeria monocytogenes* [20]. For example, the cell structure of *L. monocytogenes* was disrupted by Cur-PDI, by induction of oxidative stress and protein degradation, thereby killing >99% of the pathogen cells in fresh-cut pears, while maintaining their sensory quality [17]. However, the low water solubility and chemical stability of Cur have seriously limited its application in food PDI processing [21].

β-Cyclodextrin (β-CD), a cyclic oligosaccharide, is composed of seven α-D-glucopyranoside residues linked by α-1,4-glycosidic bonds [22]. Stable inclusion complexes can be formed inside the “doughnut” structure of β-CD, which binds low molecular weight (200–800 g/mol) hydrophobic guest molecules, greatly increasing the water solubility and dietary bioavailability of the guest molecules [23]. Zein/hydroxypropyl-β-cyclodextrin nanoparticles were synthesized using a combination of antisolvent coprecipitation and electrostatic interaction, forming Cur inclusion complexes [24]. The nanoparticles had high encapsulation efficiency (89%) for Cur, increasing both its antioxidant capacity (3.6-fold) and photostability.

In our previous study, a curcumin-β-cyclodextrin (Cur-β-CD) complex was prepared as a novel photosensitizer [25]. Furthermore, a photodynamic antimicrobial film based on Cur-β-CD complex was developed and the mechanical properties, biodegradability and antimicrobial properties characterized, indicating that the film had great potential as an antimicrobial coating [26]. In this study, the actual performance of the film was assessed for preservation of chilled pork.

## 2. Materials and Methods

### 2.1. Materials

Chilled pork was obtained from a local supermarket (Fuzhou, China). Thiobarbituric acid was obtained from Shanghai Yuanye Biotechnology (Shanghai, China). Potassium chloride was obtained from Sinopharm Chemical Reagents (Shanghai, China). Plate count agar (PCA) was obtained from Beijing Solarbio Technology (Beijing, China). Boric acid and other reagents were obtained from Shanghai Jinshan Chemical Reagents (Shanghai, China) or Aladdin Biochemical Technology (Shanghai, China). All chemicals were of analytical grade.

### 2.2. Preparation of the Cur-β-CD Photodynamic Antibacterial Film

The Cur-β-CD complex was prepared based on our previously reported method [25]. In brief, 400 mg of β-cyclodextrin and curcumin solution were mixed together and then stirred in the dark for 24 h. After centrifugation (1000 rpm, 5 min), the supernatant was freeze-dried to obtain Cur-β-CD.

Cur-β-CD photodynamic antibacterial film (1% PS-CF) was prepared as described previously [26] In brief, κ-Car powder (1%, *w*/*v*) and glycerol (50% *w*/*w* of dry weight of κ-Car) were dissolved in sterile water at 80 °C. The film-forming solution (FFS) was then obtained by magnetic stirring for 30 min. Subsequently, the Cur-β-CD (1%, *w*/*v* of FFS) was dissolved. Finally, the solution was poured into the mold and dried at 24 °C for 48 h.

### 2.3. Application of Photodynamic Antibacterial Film onto Chilled Pork

The chilled pork was trimmed to remove visible connective tissue and fat, and then cut into cubes weighing 25 ± 5 g. The pork was wrapped with conventional plastic wrap, pure carrageenan film (CF), or photodynamic antibacterial film (1% PS-CF). Three replicate pork cubes were subjected to each of four treatments: (1) conventional plastic wrap, no light treatment (control); (2) CF wrap with light treatment (wavelength at 425 nm) for 45 min (PS-L+); (3) PS-CF wrap, no light treatment (PS+L-); (4) PS-CF wrap, with light treatment (wavelength at 425 nm) for 45 min (PS+L+). All test samples were stored at 4 ± 1 °C and 55–65% humidity (ambient humidity in the refrigerator). Samples were taken every two days.

### 2.4. Evaluation of Effectiveness of Photodynamic Antibacterial Film

#### 2.4.1. Total Viable (Bacterial) Count (TVC) Determination

The TVC of pork samples was determined to evaluate microbial growth. Briefly, on the day of sampling, 5 g of the pork sample was placed in a sterile homogenizing bag and homogenized at 8000 rpm for 2 min with 45 mL of 0.9% sterile physiological saline, resulting in a 10-fold dilution, which was serially diluted as required for analysis. An aliquot (0.1 mL) of the appropriate dilution was inoculated onto plate count agar medium and incubated at 37 °C for 48 h.

#### 2.4.2. Sensory Evaluation

The sensory evaluation of pork samples was performed as described previously [27] with minor modifications. The sensory panel was composed of 10 trained individuals from the College of Food Science, Fujian Agriculture and Forestry University. The panelists were trained over three sessions to familiarize them with the attributes to be evaluated. Chilled pork samples, after the different treatments and from different storage intervals, were unwrapped and provided to each panelist separately and the color, odor, surface stickiness and overall acceptability were assessed. All indicators were scored on a 10-point scale (Table 1); a score on any criterion of 1–2 was considered as “unacceptable”.

#### 2.4.3. pH

A pork sample (10 g) was placed in a sterile homogenizing bag, distilled water (100 mL) added, then homogenized (Scientz-09, Scientz, Ningbo, Zhejiang) at 10,000 rpm for 1 min. The pH of the homogenate was determined with a PB-10 pH meter (Sartorius, Beijing, China).

#### 2.4.4. Total Volatile Basic Nitrogen (TVB-N)

TVB-N was measured using the semi-micro Kjeldahl method [28]. In brief, 10 g of pork sample was homogenized with 50 mL of distilled water at 10,000 rpm for 1 min. The homogenate was filtered before use; 5 mL of the filtrate was mixed with 5 mL of 1% MgO solution and then distilled for 5 min. Subsequently, the collected distillate was mixed with 10 mL of 2% boric acid containing methyl red-bromocresol green mixed indicator. Then, the obtained solution was titrated with 0.01 mol/L of hydrochloric acid. The TVB-N value was expressed as mg/100 g sample.

#### 2.4.5. Thiobarbituric Acid Reactive Substances (TBARS) 

The TBARS assay was conducted to evaluate lipid peroxidation, as described previously [29,30,31], with minor modifications. Briefly, a pork sample (5 g) was homogenized, as above, with distilled water (20 mL) and trichloroacetic acid solution (25 mL, 7.5% (*w*/*v*)) for 1 min, then left to stand for 30 min at room temperature. After filtering, the volume was adjusted to 50 mL by adding distilled water, then a 10 mL aliquot mixed with TBARS solution (10 mL, 0.02 mol/L). The solution was heated in a boiling water bath for 40 min, cooled to room temperature and the absorbance at 532 nm was measured by UV spectrophotometry (UV-1100, Mapada, Shanghai, China) in three parallel sets. TBA was calculated (absorbance × 7.8) and expressed as mg malondialdehyde (MDA) kg^−1^ of pork.

#### 2.4.6. Water-Holding Capacity (WHC)

WHC was measured as described in [27], with minor modifications. The surface of pork samples (~10 g) was dried with absorbent paper, then weighed (*M_1_*). The samples were then wrapped in blotting paper and centrifuged at 10,000 g for 15 min and reweighed (*M_2_*). The WHC was calculated as follows:

(1) $$WHC\;\left(\%\right)=\frac{M_{2}}{M_{1}}\times 100\%$$

#### 2.4.7. Color

After absorbing excess water from the surface of pork samples with absorbent paper, the CIELAB color parameters were determined with a colorimeter (CR-400, Konica Minolta, Tokyo, Japan). A standard white plate was used as a control and the brightness (L*), and redness (a*) were recorded. Each sample was measured five times in parallel by taking measurements in different locations.

#### 2.4.8. Hardness

The hardness of each sample was determined using a texture analyzer (EZ-test, Shimadzu, Kyoto, Japan). The instrument parameters used for testing were: P36 cylindrical probe; pre-test speed, 3.0 mm/s; test speed, 1.0 mm/s; post-test speed, 3.0 mm/s; test distance, 4 mm.

#### 2.4.9. Statistical Analysis

All analyses were performed at least in triplicate and data were expressed as mean ± standard deviation (SD). Experimental data were analyzed using DPS software (Hangzhou RuiFeng Information Technology, Co., Ltd., Hangzhou, China) and Duncan’s multiple comparison test was used for analysis of differences, with *p* < 0.05 considered significant. Origin software was used for graphing.

## 3. Results

### 3.1. Variation in TVC of Chilled Pork during Storage

**Figure 1:**
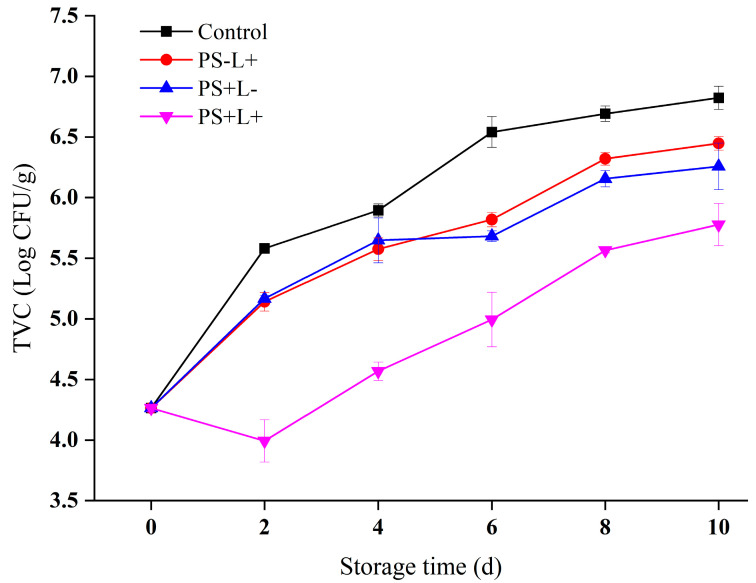
Changes in the total viable counts (TVC) in pork stored at 4 °C for 10 days. Data are presented as mean ± SD of triplicate determinations. Control (plastic wrap/no light), PS-L+ (CF wrap/light), PS+L- (PS-CF wrap/no light) and PS+L+ (PS-CF wrap/light).

Microbial growth is the main factor causing spoilage of chilled pork. The total viable count (TVC) indicates the degree of bacterial contamination of chilled pork and reflects whether the food is spoiled from excessive microbial growth. The generally accepted TVC threshold at which chilled pork is considered spoiled and unfit for human consumption is 6.00 log CFU/g [32]. The initial microbial count of the fresh pork was 4.26 ± 0.03 log CFU/g, well below the spoilage threshold (Figure 1). The TVC under all treatments increased with storage time; an increased TVC during the storage of chilled pork results from the proliferation of psychrophiles (cold-tolerant bacteria) [33]. The TVC of the control increased most rapidly, reaching 6.54 ± 0.13 log CFU/g on day 6, exceeding the spoilage threshold.

The TVC of PS+L- pork (PS-CF wrap, no light treatment) exceeded the threshold by the 8th day. Cur appears to have antimicrobial effects, even without light treatment; it interacts with numerous molecular targets and signal transduction pathways, thereby inhibiting microbial growth and proliferation. Cur can also disrupt cell membrane integrity, increasing permeability and causing leakage of cytoplasm, thereby inducing cell-death [34]. In addition, Cur downregulates bacterial gene expression, inhibits the bacterial response to DNA-damage and interacts directly with DNA to achieve bacteriostatic effects [35].

The TVC of PS-L+ pork (CF wrap, with light treatment) increased at a similar rate to that of PS+L-. Even with no Cur present, light treatment appears to have antimicrobial effects, due to the fact that the bacterial metabolism produces large amounts of endogenous porphyrins, which combine with triplet oxygen to form unstable singlet oxygen (a free radical) in the presence of light, increasing cellular oxidative stress. Oxidative stress inhibits cell growth and induces cell death [36].

The TVC value of PS+L+ pork (PS-CF wrap, with light treatment) on day 2 (3.99 ± 0.17 log CFU/g) was lower than the initial value on day 0 (4.26 ± 0.03 log CFU/g), then the TVC increased to 5.78 ± 0.18 log CFU/g on day 10, still below the spoilage threshold, indicating that PS+L+ had the highest antimicrobial activity. Compared with photodynamic antibacterial film or light treatment alone, Cur-mediated photodynamic inactivation (PDI) significantly inhibited microbial growth and proliferation, extending the shelf life of the chilled pork from 4–5 days to 10 days. These results are in agreement with a previous report [17], that Cur-mediated PDI resulted in a >5 log CFU/g reduction in the TVC of *L. monocytogenes* compared with light or Cur alone.

The water solubility of Cur increases 31-fold when it forms the Cur-β-CD complex, which appears to greatly enhance the photodynamic bactericidal effect [37]. Similarly, the antimicrobial activity of Cur is greatly increased by formation of the (2-Hydroxy isopropyl)-β-cyclodextrin (HPβCD) complex. The TVCs of methicillin-resistant *Staphylococcus aureus* and *Pseudomonas aeruginosa* have both been shown to decrease by >87% due to the action of alginate hydrogels containing HPβCD-Cur, compared with Alg/Cur (<69%) [38]. Cur-β-CD causes microbial cell deformation, surface collapse and membrane disruption, resulting in leakage of cytoplasm [25]. PDI mediated by photodynamic antibacterial film was an effective way to reduce the TVC of chilled pork during refrigerated storage, slowing spoilage and markedly extending its shelf life.

### 3.2. pH Variation in Chilled Pork during Storage

**Figure 2:**
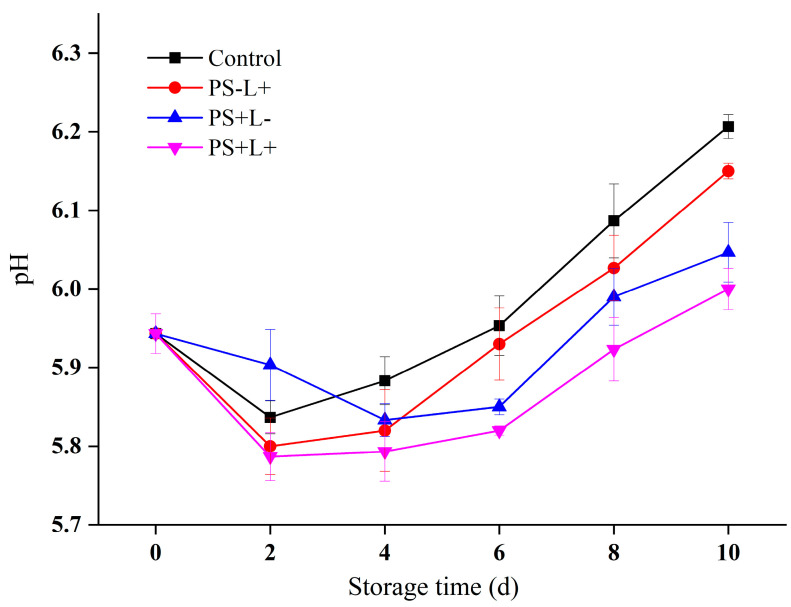
Changes in pH of pork stored at 4 °C for 10 days. Data are presented as mean ± SD of triplicate determinations. Control (plastic wrap/no light), PS-L+ (CF wrap/light), PS+L- (PS-CF wrap/no light) and PS+L+ (PS-CF wrap/light).

pH is an important indicator of chilled pork freshness. The initial pH value of the pork was 5.94 ± 0.03 (Figure 2), then the pH under all treatments decreased to a minimum at 2–4 days, then increased until the end of the cold storage period, consistent with a previous report [32]. Initially, muscle glycogen is converted to lactic acid after slaughter, as the respiration of the pork changes from aerobic to anaerobic, combining with phosphate from breakdown of adenosine triphosphate, to decrease the pH. During chilled storage, the muscle proteins are broken down by both endogenous and microbial proteases, producing alkaline species such as ammonia and amines [39], increasing the pH. Compared with the control pork, the pH of the PS+L- and PS-L+ treatments was lower through most of the storage period, indicating that the individual antimicrobial effects of Cur and light inhibited the growth of spoilage bacteria and inhibited the pH increase. In addition, the κ-Car film used in both treatments has good barrier properties that should inhibit the protein decomposition, lipid oxidation and lipid spoilage resulting from the accelerated microbial growth. A similar reduction in meat pH resulting from Cur and light treatment was observed in chicken breast fillets and Schizothorax prenanti surimi [32,39,40]. At day 10, the pH values resulting from the PS-L+, PS+L- and PS+L+ treatments were 6.21 ± 0.02, 6.05 ± 0.01, and 6.00 ± 0.03, respectively, and the pH of PS+L+ was the lowest throughout the storage period. It appears that the PDI function of the PS+L+ film delays the pH increase by inhibiting microbial growth, in agreement with a previous report [41].

### 3.3. Variation in Total Volatile Basic Nitrogen (TVB-N) of Chilled Pork during Storage

**Figure 3:**
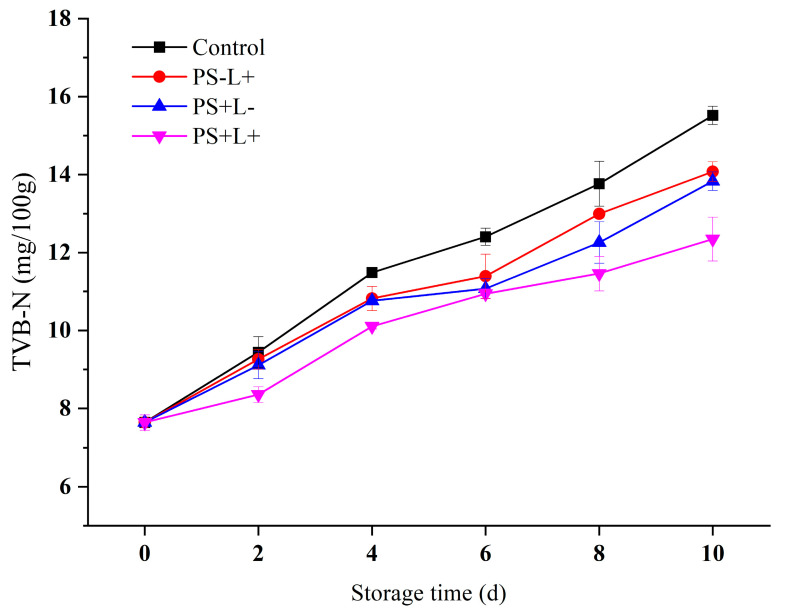
Changes in total volatile basic nitrogen (TVB-N) in pork stored at 4 °C for 10 days. Data are presented as mean ± SD of triplicate determinations. Control (plastic wrap/no light), PS-L+ (CF wrap/light), PS+L- (PS-CF wrap/no light) and PS+L+ (PS-CF wrap/light).

Meat proteins are subjected to the action of endogenous and microbial proteases, producing large amounts of alkaline nitrogenous volatiles such as ammonia and amines which contribute to the off-smell of spoiled meat [42]. These volatiles, together with other nitrogenous compounds, constitute TVB-N. Therefore, TVB-N is an important indicator of the quality and freshness of pork. The maximum acceptable TVB-N standard value for fresh livestock products is 15 mg/100 g meat [43]. The initial TVB-N value of the pork was 7.64 ± 0.20 mg/100 g (Figure 3), which is in agreement with a previous report [44]. The TVB-N after all treatments gradually increased during refrigerated storage, indicating slow but progressive proteolysis of muscle proteins. The degradation of muscle cell structures could result in the release of endogenous enzymes, which would accelerate protein degradation [43]. The control TVB-N value reached 15.52 ± 0.23 mg/100 g after 10 days, just exceeding the standard limit, whereas those of PS-L+, PS+L- and PS+L+ were 14.08 ± 0.25, 13.83 ± 0.24 and 12.34 ± 0.57 mg/100 g, respectively. These results confirm that the PDI function of the PS+L+ film delayed the TVB-N increase and maintained the TVB-N below that of the control (*p* < 0.05) and below the standard limit, by inhibiting microbial growth and muscle protein proteolysis.

### 3.4. Variation in Thiobarbituric Acid Reactive Substances (TBARS) of Chilled Pork during Storage

**Figure 4:**
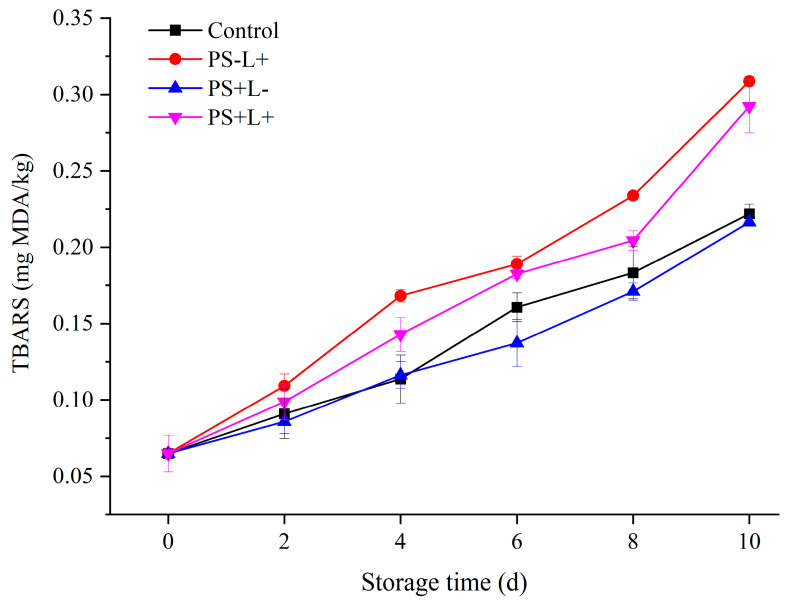
Changes in thiobarbituric acid reactive substances (TBARS) in pork stored at 4 °C for 10 days. Data are presented as mean ± SD of triplicate determinations. Control (plastic wrap/no light), PS-L+ (CF wrap/light), PS+L- (PS-CF wrap/no light) and PS+L+ (PS-CF wrap/light).

Oxidation of fat produces an unpleasant, rancid taste and harmful oxidation products such as hydroperoxides and aldehydes such as malondialdehyde (MDA). TBARS measurements reflect the degree of lipid oxidation in pork samples [45]. The TBARS values of all samples gradually increased with storage time (Figure 4), similarly to those in stored shrimp [46], due to the accumulation of MDA and other products produced by breakdown of peroxides produced during the oxidation of polyunsaturated fatty acids. Consumers can usually detect off-flavors in foods when the TBARS value exceeds the threshold of ~0.6 mg MDA/kg. The slower increase in TBARS for PS+L- compared with control may result from the antioxidant activity of Cur and is consistent with a previous report [47]. The TBARS value of PS-L+ and PS-L+ was higher than the control during storage, indicating greater lipid oxidation, which suggests acceleration of lipid oxidation by the light treatment; however, even PS-L+ was well below the 0.6 mg/kg threshold at day 10 (Figure 4). Naturally occurring photosensitizers in meat—such as tryptophan and tyrosine residues in proteins, or riboflavin—may either react directly with lipids and proteins or react with molecular oxygen, producing superoxide or singlet oxygen, both of which induce oxidative damage. Higher light intensity (>4094 lx) and longer light exposure time (>24 h) rapidly increases the oxidation rate in milk [48], but excessive oxidation can be avoided by controlling the light treatment time. Therefore, when using PDI film for meat preservation, the irradiation time and light intensity should be carefully controlled to achieve optimal preservation.

### 3.5. Variation in Water-Holding Capacity (WHC) of Chilled Pork during Storage

**Figure 5:**
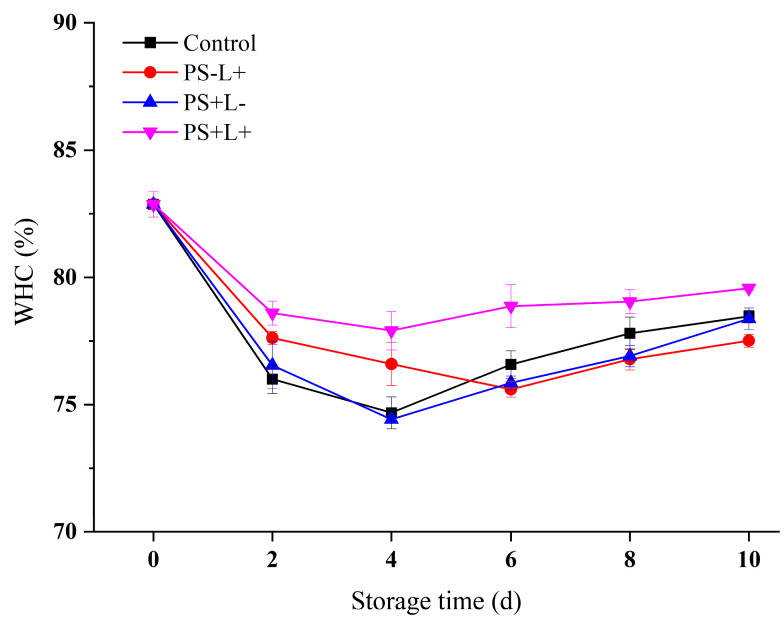
Changes in water-holding capacity (WHC) in pork stored at 4 °C for 10 days. Data are presented as mean ± SD of triplicate determinations. Control (plastic wrap/no light), PS-L+ (CF wrap/light), PS+L- (PS-CF wrap/no light) and PS+L+ (PS-CF wrap/light).

WHC is a measure of the ability of meat to retain water and minimize drip-loss [49]; meat with poor WHC has a dry, rough texture and high weight-loss [50]. The WHC values of all meat samples initially decreased rapidly, to a minimum after ~4 days of storage, followed by a gradual increase (Figure 5). WHC is related to the net charge on protein molecules, which changes with pH value; generally, the higher the pH of meat, the greater the net negative charge on the meat proteins and the greater the WHC [51]. The variation in WHC with time is very similar to that of pH (Figure 2), indicating that pH was a major determinant of WHC in the stored pork. However, the WHC of PS+L+ was the highest throughout the chilled storage period, even though its pH was the lowest during storage (Figure 2), so additional factors must be involved. It appears that the antimicrobial effect of the PDI film inhibited microbial growth and protease production, thereby decreasing muscle protein degradation and increasing the relative WHC. The WHC value of PS-L+ was lower than control from days 6 to 10 of storage, apparently because light treatment accelerated protein oxidation and exposed their hydrophobic amino acid side-chains, resulting in a lower WHC.

### 3.6. Color Variation of Chilled Pork during Storage

Color is an important visual indicator of meat freshness, with a direct impact on consumer acceptance and purchasing decisions. The color of meat after slaughter is not only related to its myoglobin content and the oxidation state of the myoglobin, but also to the degree of lipid oxidation [52]. The brightness (L*) values of all samples initially increased moderately, peaking at day 4 of storage (Table 2), indicating that the meat became lighter in color. The L* values then decreased until day 10. The L* value for PS+L+ on day 10 (43.13 ± 1.80) was very close (*p* > 0.05) to the initial value on day 0, suggesting that the PDI-film prevented the darkening of the pork, which was observed with the other treatments. The a* (redness) values of all samples increased until day 2 or 4, then decreased (Table 3), which is related to the degree of myoglobin oxygen-binding. The a* values initially increased, because of the formation of red, highly-oxygenated myoglobin in the pork [53], then decreased after day 4 due to oxidation of the myoglobin to brown metmyoglobin. The a* value of PS-L+ decreased the fastest, probably because lipid oxidation was accelerated by the light treatment. The a* values of PS+L- and PS+L+ increased similarly to those of the control and PS-L+ until day 4, but decreased much more slowly and were markedly higher at day 10, indicating that the antioxidant activity of Cur in the former treatments inhibited lipid oxidation and the oxidative browning of myoglobin. Phenolic compounds inhibit cellular production of peroxides and free radicals [54] and delay myoglobin oxidation to metmyoglobin [33]. It appears that PS-CF film effectively delays myoglobin oxidation in chilled pork by controlled release of Cur onto the meat’s outer surface.

### 3.7. Variation in the Hardness of Chilled Pork during Storage

Hardness is an objective indicator of the textural characteristics and quality of chilled pork, and a predictor of consumer acceptance. The hardness values of chilled pork after all treatments generally increased until day 6 or 8 of storage, then decreased (Table 4). The muscle tissue enters rigor mortis after slaughter, because the muscles can no longer relax when the supply of ATP is exhausted, markedly decreasing muscle elasticity and increasing its hardness. The subsequent decrease in meat hardness/rigor is caused by proteolytic autolysis of the muscle proteins, combined at the later stage of storage with microbial proteolysis [55,56,57]. The decrease in hardness values of PS-L+, PS+L- and PS+L+ was slower than in the control, partly because the carrageenan film had higher air permeability than the plastic wrap, allowing gas exchange with the outside environment and greater water loss from the pork. In addition, the antioxidant and antimicrobial activities of PS-CF film would have inhibited microbial proteolysis and oxidation, delaying muscle protein degradation and the hardness decrease. The hardness of PS+L- and PS+L+ was still significantly higher at day 10 of storage than at day 0, whereas the hardness of PS-L+ was much higher and that of the control was significantly lower.

### 3.8. Sensory Evaluation of Chilled Pork during Storage

**Figure 6:**
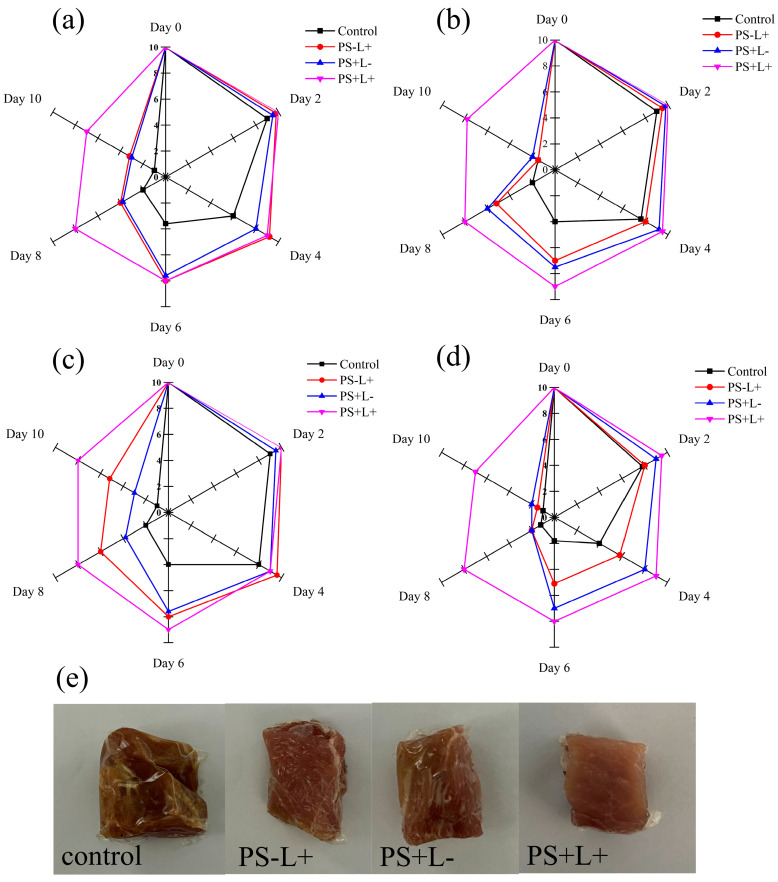
Changes in the sensory evaluation of chilled pork in the control group, PS-L+ group, PS+L- group and PS+L+ group at 4 °C for 10 days. (**a**) color score; (**b**) odor score; (**c**) viscosity score; (**d**) acceptability score; (**e**) appearance.

The smell, color and surface stickiness of chilled pork are important determinants of consumer acceptability. Darkening/browning of the color and the increasingly unpleasant smell of pork during chilled storage mainly result from microbial spoilage and lipid oxidation. The color, odor, viscosity and overall acceptability scores of all treatments decreased during storage, with the control “just acceptable” at day 6 and “unacceptable” by day 8 (Figure 6). The control had a greyish-brown color and a strong putrid smell at days 8 and 10, however, PS+L+ scored “just acceptable” or “moderately acceptable” at day 10 and markedly higher than control throughout the storage period. Given that the control had an unacceptably high TVC value at day 6 (>6 log CFU/g), whereas that of PS+L+ was well below the threshold of unacceptability (Figure 1), even at day 10, it is clear that the control shelf-life was no more than 4–5 days, whereas that of PS+L+ was 10 days.

### 3.9. Correlation Analysis of Pork Quality Indicators

**Figure 7:**
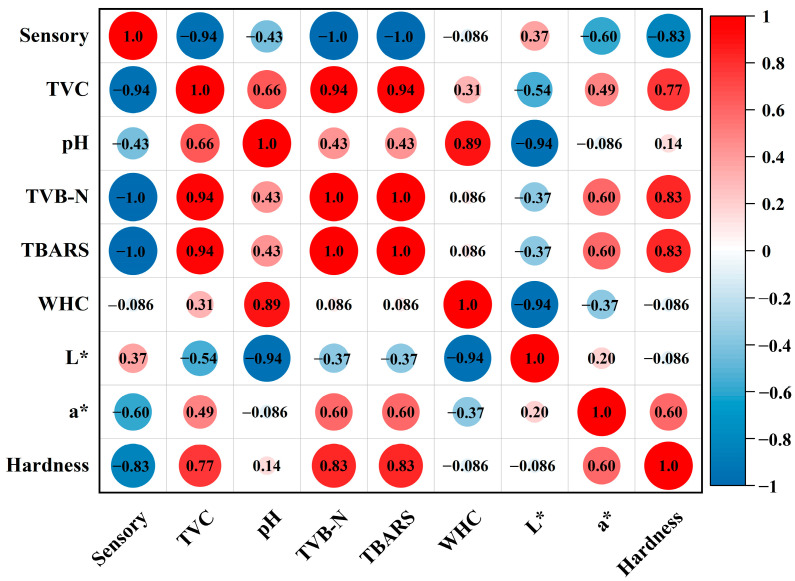
Correlation analysis between various indicators of chilled pork. Red means positive correlation, blue means negative correlation. The darker the color meant the stronger correlation between the two indicators, while the lighter meant the weaker correlation.

Correlation analysis of the quality indicators of the samples treated with PS+L+ and stored at 4 °C was performed by calculating Pearson’s correlation coefficients (r) (Figure 7). The sensory scores were negatively correlated with TVC, TVB-N and TBARS (R = −0.943, −1 and −1, respectively; *p* < 0.01) and positively correlated with hardness (R = −0.828, *p* < 0.05). TVB-N and TBARs showed a significant positive correlation with TVC, i.e., they are good indicators of meat spoilage and from a practical perspective they are much simpler to perform than TVC. The sensory scores of chilled pork decreased with increasing values of TVC, hardness, TVB-N and TBARS, and there was a significant positive correlation between TVC, TVB-N, TBARS and hardness (*p* < 0.05), indicating that TVB-N, TBARS and TVC are good indicators of chilled pork quality.

## 4. Conclusions

In this study, photodynamic (PDI) antibacterial carrageenan film was applied to extend the shelf life of chilled pork. The PDI film significantly inhibited the microbial growth and lipid oxidation of the pork during storage, increased the WHC and improved the color. These changes markedly improved the sensory attributes of the chilled pork, thereby extending its shelf life from 4–5 to 10 days. In addition, there were strong correlations between the sensory scores of the chilled pork and TVC, hardness, TVB-N and TBARS. However, only the meat preservation effect of the PDI film was investigated in this study; future research should determine whether curcumin migrates from the film into the meat, the rate of any migration, and the mechanisms of any direct effects on meat preservation and quality.

## Figures and Tables

**Table 1:** Sensory evaluation criteria.

Criterion	Score Range				
	9~10	7~8	5~6	3~4	1~2
Color	bright red, shiny	red, shiny	darker red	dull, dark red	dull grey-brown, white spots
Odor	normal pork odor	light meaty, no off odor	mild off odor	strong off odor	putrid odor
Surface stickiness	not sticky	slightly sticky	moderately sticky	sticky	very sticky
Overall acceptability	good	acceptable	moderately acceptable	just acceptable	unacceptable

**Table 2:** Changes in L* (brightness) value of pork stored at 4 °C for 10 days.

Time/d	0	2	4	6	8	10
Control	43.41 ± 1.26 ^Ab^	48.70 ± 2.67 ^Aa^	48.00 ± 0.25 ^Aa^	46.49 ± 1.71 ^Aa^	42.61 ± 1.74 ^Ab^	40.49 ± 0.91 ^ABb^
PS-L+	43.41 ± 1.26 ^Aab^	44.28 ± 2.81 ^Aab^	45.56 ± 0.45 ^Aa^	43.97 ± 0.65 ^Bab^	42.07 ± 1.26 ^Ab^	38.23 ± 2.65 ^Bc^
PS+L-	43.41 ± 1.26 ^Aab^	47.52 ± 1.93 ^Aa^	47.44 ± 2.47 ^Aa^	46.54 ± 0.37 ^Aa^	43.93 ± 3.71 ^Aab^	40.91 ± 1.78 ^ABb^
PS+L+	43.41 ± 1.26 ^Ab^	46.5 ± 1.28 ^Aa^	47.19 ± 1.19 ^Aa^	45.88 ± 0.94 ^ABa^	45.72 ± 1.00 ^Aa^	43.13 ± 1.80 ^Ab^

Different letters in the same column or row (uppercase and lowercase columns and rows, respectively) indicate a significant difference (*p* < 0.05).

**Table 3:** Changes in a* (redness) value of pork stored at 4 °C for 10 days.

Time/d	0	2	4	6	8	10
Control	3.9 ± 0.69 ^Ab^	6.36 ± 0.33 ^Aa^	6.57 ± 2.06 ^Aa^	4.06 ± 0.73 ^Bb^	4.76 ± 0.59 ^BCab^	3.7 ± 0.24 ^Bb^
PS-L+	3.9 ± 0.69 ^Acd^	5.31 ± 0.45 ^Ab^	6.49 ± 0.53 ^Aa^	4.27 ± 0.76 ^ABc^	4.17 ± 0.23 ^Ccd^	3.16 ± 0.59 ^Bd^
PS+L-	3.9 ± 0.69 ^Ab^	6.88 ± 1.53 ^Aa^	6.59 ± 0.18 ^Aa^	6.08 ± 1.44 ^Aab^	7.55 ± 2.06 ^Aa^	5.31 ± 0.61 ^Aab^
PS+L+	3.9 ± 0.69 ^Ac^	5.69 ± 0.14 ^Ab^	6.21 ± 0.4 ^Aab^	4.63 ± 0.52 ^ABc^	6.59 ± 0.32 ^ABa^	5.93 ± 0.28 ^Aab^

Different letters in the same column or row (uppercase and lowercase columns and rows, respectively) indicate a significant difference (*p* < 0.05).

**Table 4:** Changes in the hardness of pork stored at 4 °C for 10 days.

Time/d	0	2	4	6	8	10
Control	247.96 ± 9.53 ^Aab^	263.50 ± 17.89 ^Aab^	255.80 ± 37.62 ^Aab^	295.68 ± 87.82 ^Aa^	261.00 ± 38.94 ^Bab^	202.36 ± 22.64 ^Bb^
PS-L+	247.96 ± 9.53 ^Ac^	325.49 ± 47.31 ^Abc^	316.41 ± 71.84 ^Abc^	376.83 ± 8.62 ^Ab^	482.73 ± 84.74 ^Aa^	389.14 ± 52.5 ^Aab^
PS+L-	247.96 ± 9.53 ^Ab^	265.70 ± 71.16 ^Aab^	246.53 ± 37.14 ^Ab^	331.51 ± 48.64 ^Aa^	296.91 ± 35.57 ^Bab^	284.87 ± 19.27 ^Bab^
PS+L+	247.96 ± 9.53 ^Aa^	260.81 ± 39.49 ^Aa^	264.18 ± 69.93 ^Aa^	291.81 ± 81.84 ^Aa^	309.74 ± 67.43 ^Ba^	282.42 ± 73.98 ^Ba^

Different letters in the same column or row (uppercase and lowercase columns and rows, respectively) indicate a significant difference (*p* < 0.05). The hardness value was expressed as N.

## Data Availability

Data available on request.
